# Paired Stimulation of Different Digits for 30 min Does Not Produce Long-Term Plastic Changes in the Human Cutaneomuscular Reflex

**DOI:** 10.1523/ENEURO.0103-24.2024

**Published:** 2025-03-19

**Authors:** Maria Germann, Eldesta Nabila, Stuart N. Baker

**Affiliations:** Faculty of Medical Sciences, Newcastle University, Newcastle upon Tyne NE2 4HH, United Kingdom

**Keywords:** cutaneomuscular reflex, spike-timing–dependent plasticity

## Abstract

Cutaneomuscular reflexes (CMRs) can be recorded in the hand muscle of human subjects after stimulation of a digital nerve. We hypothesized that repeated synchronous stimulation of nerves from two digits may lead to long-term plastic changes in CMR, by the mechanisms of spike-timing–dependent plasticity (STDP). To test this idea, we conducted experiments in 27 healthy human volunteers. After baseline measurement of CMR, one of four 30-min-long stimulation conditions were tested; the CMR was then remeasured. The four conditions were simultaneous index finger and thumb stimulation; asynchronous index finger and thumb stimulation; thumb 5 ms before index finger stimulation; and thumb-only stimulation. Neither the early (E1) nor late excitatory (E2) components of the CMR showed consistent changes after any stimulation condition. The inhibitory (I1) component was slightly reduced in all cases. To understand why paired stimulation did not produce long-term changes, we conducted a further experiment. In this, we measured the CMR in response to simultaneous stimulation of index finger and thumb, compared with a prediction expected if the responses summed linearly. This revealed sublinear summation, possibly indicating partial response saturation after stimulation of only one digit. We argue such a pattern prevents paired stimuli from generating especially reliable and well-timed outputs relative to synaptic inputs in downstream neurons, which is required to produce plasticity by STDP.

## Significance Statement

Cutaneomuscular reflexes (CMRs) were recorded in the hand muscle of human subjects after repeated synchronous stimulation of two digital nerves. Surprisingly, we found no evidence of long-term plastic changes in the CMR after paired stimulation. Comparison of the CMR responses to stimuli given to one digit alone versus both digits together revealed sublinear reflex summation, which could indicate partial response saturation after stimulation of only one digit. We argue such a pattern prevents paired stimuli from generating reliable and well-timed outputs relative to synaptic inputs in downstream neurons, which is required to produce plasticity by spike-timing–dependent plasticity.

## Introduction

Neuronal connections within the motor system have the capacity to reorganize and modify, based on the input they receive. Neuromodulation strategies have been developed to exploit these mechanisms to induce long-term plastic changes in motor circuits. Such approaches may have promise in enhancing recovery following damage to the motor system, such as after stroke or spinal cord injury.

In humans, many noninvasive stimulation protocols have been proven to generate plasticity in the motor cortex and its corticospinal projections. These include stimulation of the motor point of two muscles (Ridding and Uy, 2003; Pyndt and Ridding, 2004; McDonnell and Ridding, 2006; Schabrun and Ridding, 2007), of two peripheral nerves (Ridding et al., 2000; 2001; McKay et al., 2002; Charlton et al., 2003), of the motor cortex alone (Pascual-Leone et al., 1994), or of the motor cortex combined with peripheral nerve stimulation (Stefan et al., 2000; Ridding and Taylor, 2001; Quartarone et al., 2003; Ridding and Flavel, 2006; Taylor and Martin, 2009; Bunday et al., 2018). Approaches using paired stimuli typically rely on the activated inputs converging onto a common target neuron (Brown et al., 2016). This can generate plasticity via the principle of spike-timing–dependent plasticity (STDP; Markram et al., 1997).

Compared with the wealth of studies on the motor cortex, relatively few publications report evidence for the induction of plasticity in subcortical systems. Pairing loud click sounds with peripheral stimuli can generate plastic changes, likely to be in reticulospinal circuits (Foysal et al., 2016; Germann and Baker, 2021). Pairing tone bursts, loud enough to induce a startle reflex, with transcranial magnetic stimulation (TMS) over the motor cortex, also induces plastic changes most likely to lie in corticoreticular connections (Germann et al., 2023). Additionally, there is evidence that paired stimulation of the motor cortex with peripheral nerves, intended to target cortical plasticity, can also generate long-lasting changes in spinal circuits (Meunier et al., 2007; Lamy et al., 2010).

One situation where the potential for plastic change has been little explored is the cutaneomuscular reflex (CMR). This is straightforwardly elicited by electrical stimulation of a digital nerve and results in a clearly defined reflex response in hand muscles such as first dorsal interosseous (FDI; Garnett and Stephens, 1980). The CMR response consists of three phases: an early excitation E1, followed by inhibition I1 and then a late excitation E2. Based on its short latency, E1 is believed to originate mainly within spinal circuits (Garnett and Stephens, 1980; Jenner and Stephens, 1982), while the later I1 and E2 are probably mediated by transcortical pathways (Carr et al., 1993; Mayston et al., 1997). Consistent with this, in patients with motor cortical damage, I1 and E2 are absent, but E1 is exaggerated (Jenner and Stephens, 1982).

A CMR can be induced in a given muscle by stimulation of multiple digits. This raises the interesting possibility that separate cutaneous inputs may converge onto common neurons and that repeated simultaneous activation of different digits could induce plastic changes. In this study, we sought to test these hypotheses. Surprisingly, we find no evidence for such plasticity after paired stimulation of different digits.

## Materials and Methods

### Subjects

In total, 27 healthy adult volunteers (18 females) took part in the study. Nineteen subjects (14 females) participated in the paired stimulation experiment, and seven subjects (4 females) participated in the reflex summation experiment. As there were four different paring conditions, subjects participating in the paired stimulation experiment were asked to come to four different visits, separated by at least 10 d to avoid potentially long-lasting effects influencing the next session. Of the 19 subjects participating in the paired stimulation experiment, 19 took part in the synchronous condition, 19 in the asynchronous condition, 15 in the 5 ms delay condition, and 10 in the thumb-only condition. All subjects gave written informed consent to the experimental procedures, which were approved by the local ethics committee. The study was performed in accordance with the guidelines established in the Declaration of Helsinki, except that the study was not preregistered in a database.

### Electromyography (EMG) recordings

EMG was recorded from the FDI muscle through surface electrodes secured on the skin over the muscle belly, after first cleaning the skin with an alcohol swab. EMG signals were amplified and filtered (bandpass 30–2,000 Hz) with a bioamplifier (D360 8-Channel Patient Amplifier, Digitimer) and then digitized with a sampling rate of 5 kHz (CED Micro 1401 with Spike2 software, Cambridge Electronic Design) and stored on a computer for off-line analysis.

### Electrical stimulation

**Figure 1.**
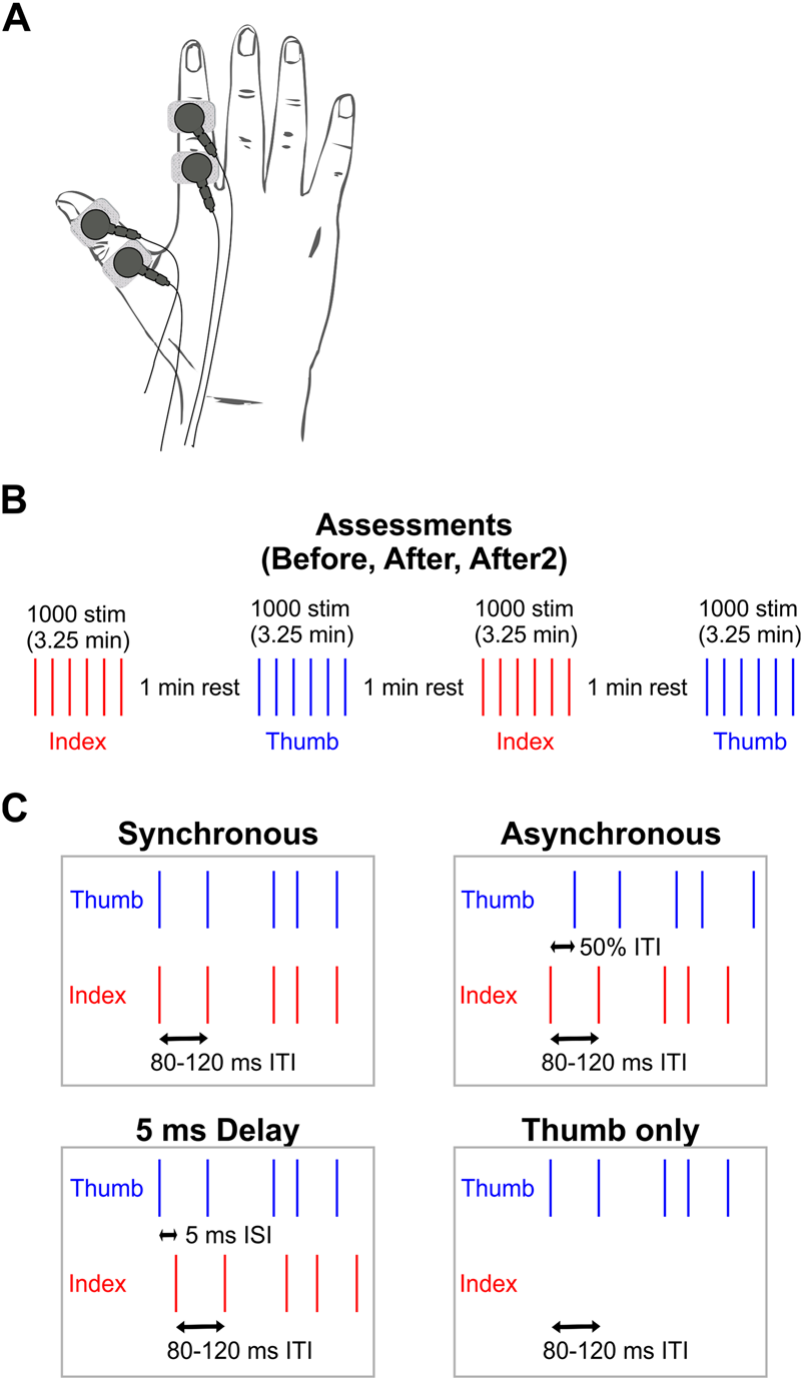
Experimental design. ***A***, Schematic representation of stimulation electrodes placement. ***B***, Stimulation protocol for the assessments carried out before, 0–18 min after, and 18–36 min after (after2). ***C***, Stimulation protocols for synchronous (top left), asynchronous (top right), 5 ms delay (bottom left), and thumb-only (bottom right) condition. ISI, interstimulus interval; ITI, intertrial interval.

Stimulation consisted of electrical stimuli generated by two constant-current stimulator devices (DS7, Digitimer). Monophasic square-wave current pulses (pulse duration, 500 μs) were delivered via two pairs of surface electrodes to the thumb and index finger, respectively (cathode proximal, anode–cathode separation∼1 cm; Fig. 1*A*).

Perceptual threshold was determined for each digit by gradually increasing the intensity until the participant could detect the stimulus. Participants were not informed in advance whether they received thumb or index finger stimulation. Stimulus intensity for the experiment was set as three times the individual's perceptual threshold.

### Paired stimulation protocols

Each protocol consisted of four sections: a baseline assessment (before), which was followed by one of the four paired stimulation protocols (intervention) and two postpairing assessments lasting 0–18 min (after) and 18–36 min (after2) after the end of the intervention. Baseline and postpairing assessments were identical for all protocols.

During the intervention, subjects were told to relax their hand. During the assessments, subjects were asked to perform an index finger abduction at 20% of their maximum voluntary contraction (MVC). Visual feedback of rectified and smoothed EMG activity from the FDI muscle was provided to the subjects, involving a series of colored bars on a computer screen which illuminated in sequence as stronger contractions were made. This system was first calibrated to the subject's individual MVC.

#### Assessments

A total of 2,000 stimuli were given to each thumb and index finger during each assessment (Fig. 1*B*). First, a block of 1,000 stimuli were delivered to the index finger, followed by 1 min of rest. Afterward a block of 1,000 stimuli was delivered to the thumb, again followed by a 1 min resting period. Both blocks were then repeated once more. Each block lasted 3.25 min and were separated by 1 min of rest.

In-between baseline and the two postpairing assessments, subjects were conditioned with one of the four paired stimulation protocols described below, each of which lasted ∼30 min and were delivered at rest. A total of 18,000 paired stimuli were given.

#### Synchronous

For synchronous stimulation, thumb and index fingers were stimulated simultaneously (Fig. 1*C*). The intertrial interval (ITI) varied between 80 and 120 ms, randomly chosen from a uniform distribution.

#### Asynchronous

For asynchronous stimulation, the ITI for the index finger stimulus remained 80–120 ms. The index finger was stimulated at the start of the ITI chosen for that trial, and the thumb was stimulated halfway through the interval (Fig. 1*C*).

#### A 5 ms delay

In situations where spike-timing plasticity generates long-term changes, the maximal effect can be produced by stimuli separated by a short delay rather than precisely synchronized (Taylor and Martin, 2009). To test whether stimuli slightly separated in time produced plasticity, we stimulated the thumb 5 ms before the index finger. The same ITI as used in other conditions (80–120 ms) was used (Fig. 1*C*).

#### Thumb only

No index finger stimulation was given. Single stimuli were delivered to the thumb at an ITI of 80–120 ms (Fig. 1*C*).

### Data analysis

Data were analyzed using MATLAB (R2017a, MathWorks). EMG traces were full-wave rectified and averaged. Each component (E1, I1, E2) of the CMR was then visually selected and analyzed individually. Mean percentage change (MPC) was determined as the average change in the mean rectified EMG, normalized as a percentage of the mean baseline period (measured over a 40 ms window before stimulation).

Statistics were calculated using IBM SPSS Statistics for Windows, version 24 (IBM). A mixed ANOVA was used to compare the effect of the repeated measures of TIME (before, after, after2) and the between-subjects factor CONDITION (synchronous, asynchronous, 5 ms delay, thumb only) on each of the components of the CMR. Sphericity was tested with Mauchly's test of sphericity. When sphericity could not be assumed, the Greenhouse–Geisser correction statistic was used.

Paired *t* tests were used to compare individual data points post hoc and reported with effect size Cohen's *d*. The Benjamini–Hochberg procedure was used to correct for multiple comparisons (Benjamini and Hochberg, 1995). The significance level was set at *p* < 0.05, and group data are presented as mean ± SD in the text.

The binomial cumulative distribution function was used to determine whether the number of subjects showing a certain change relative to the baseline (increase vs decrease) was more than expected by chance, assuming the null hypothesis that an increase and decrease were equally likely (probability of 0.5).

### Reflex summation experiment

To test whether there was linear summation of the CMR elicited from index finger and thumb stimulation, we designed a second experiment.

Eight blocks of 1,000 stimuli were given, with a 1 min resting period in-between the blocks. In each block, there were 250 stimuli of each of the 4 conditions: index finger only, thumb only, index and thumb simultaneously, and no stimulation. This led to a total of 2,000 stimuli per condition across the whole experiment. As above, stimulation intensity was set as three times the perceptual threshold, and participants were asked to activate the 1DI muscle to 20% of MVC.

We wished to compare the response to paired thumb and index finger stimulation with that expected if the two stimuli summed linearly. However, nonlinear effects in rectified EMG make this comparison not straightforward. We therefore used the approach described by Baker and Lemon (1995). A “predicted” response, assuming linear summation, was calculated as followed. Single unrectified EMG sweeps from the index finger-only trials were added point by point to single sweeps of the thumb-only trials; the resulting sum was then rectified. These traces of rectified EMG were averaged across trials to produce a simulated response, as if both stimuli had been given simultaneously and summed linearly. One problem with comparing this predicted response to an average of responses to paired stimulation is that the background EMG will be higher (because it is compiled from a combination of two trials). To compensate for this, single sweeps of unrectified EMG from the simultaneous stimulation trials were added to single sweeps from the no-stimulation trials; the summed EMG was then rectified and averaged across trials to yield the “actual” response to both stimuli given together.

In both the predicted and actual response, each component (E1, I1, E2) of the CMR was visually selected and analyzed individually as described above. Paired *t* tests were used to compare the predicted and actual response for E1, I1, and E2.

## Results

### Overall reflex changes following paired stimulation

**Figure 2.**
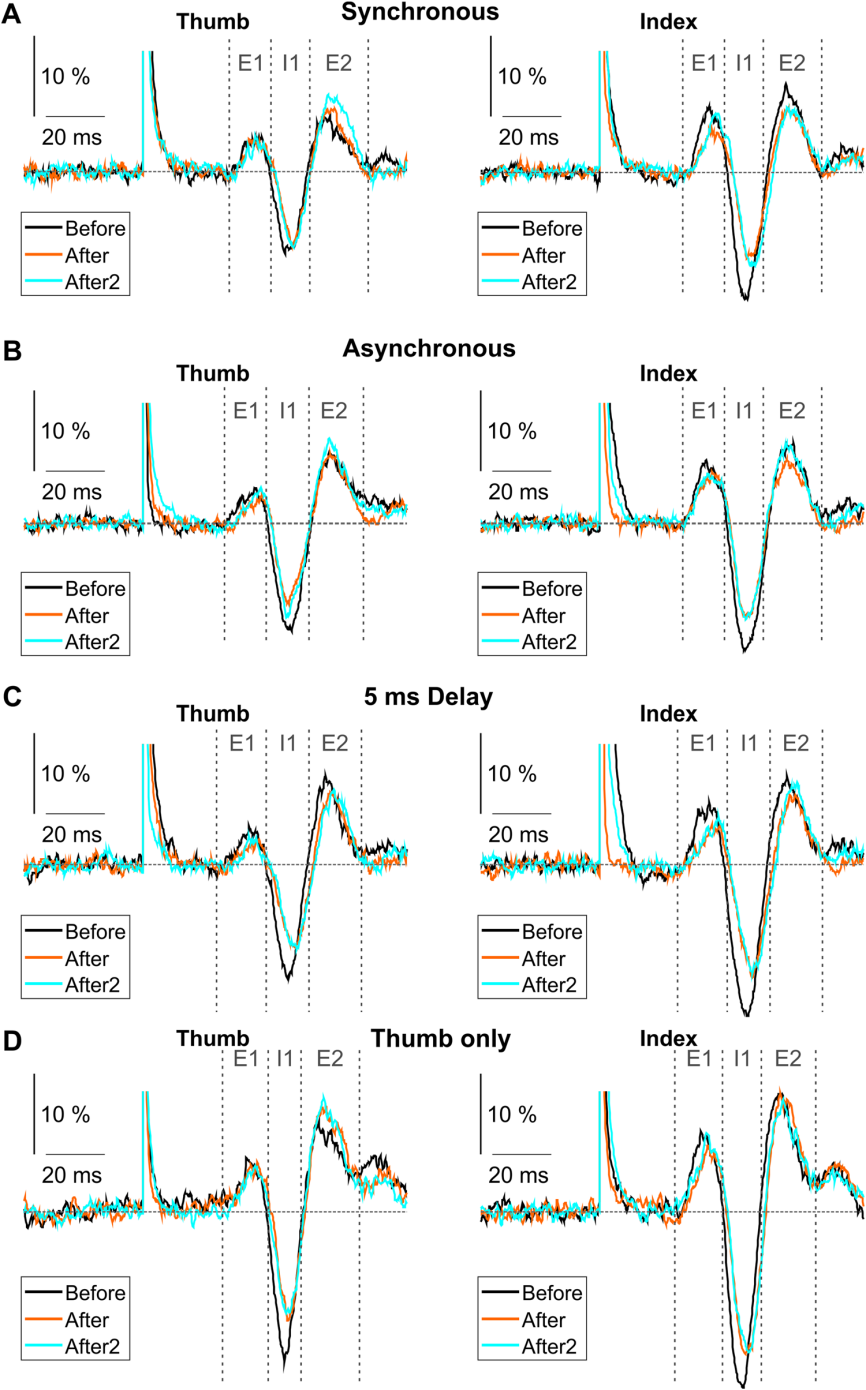
Overlay of EMG traces averaged across all subjects. The traces represent each assessment done before (black), 0–18 min after (orange), and 18–36 min after (cyan) the paired stimulation. Responses to thumb stimulation are plotted in the left column, responses to index finger stimulation are plotted in the right column. Gray vertical dotted lines indicate the three components (E1, I1, E2) of the cutaneoumuscular reflex. Gray horizontal dashed line represents the mean baseline value of the assessment done before the stimulation. The calibration bar is a percentage of the baseline EMG level. ***A***, Overlay of averaged EMG traces for the synchronous stimulation protocol. ***B***, Overlay of averaged EMG traces for the asynchronous stimulation protocol. ***C***, Overlay of averaged EMG traces for the 5 ms delay stimulation protocol. ***D***, Overlay of averaged EMG traces for the thumb-only stimulation protocol.

Figure 2 illustrates an overlay of averaged EMG traces, combined across all subjects, for the three components (E1, I1, and E2) of the CMR before, 0–18 min after, and 18–32 min after the end of the intervention (black, red, and blue traces, respectively). Separate traces are shown for assessment stimuli given to the thumb and index finger (Fig. 2, columns), and for the different paired stimulation protocols interventions (Fig. 2*A–D*, rows). It is striking how after every stimulation protocol the I1 component of the reflex was decreased.

#### Lack of changes in E1 reflex component

**Figure 3.**
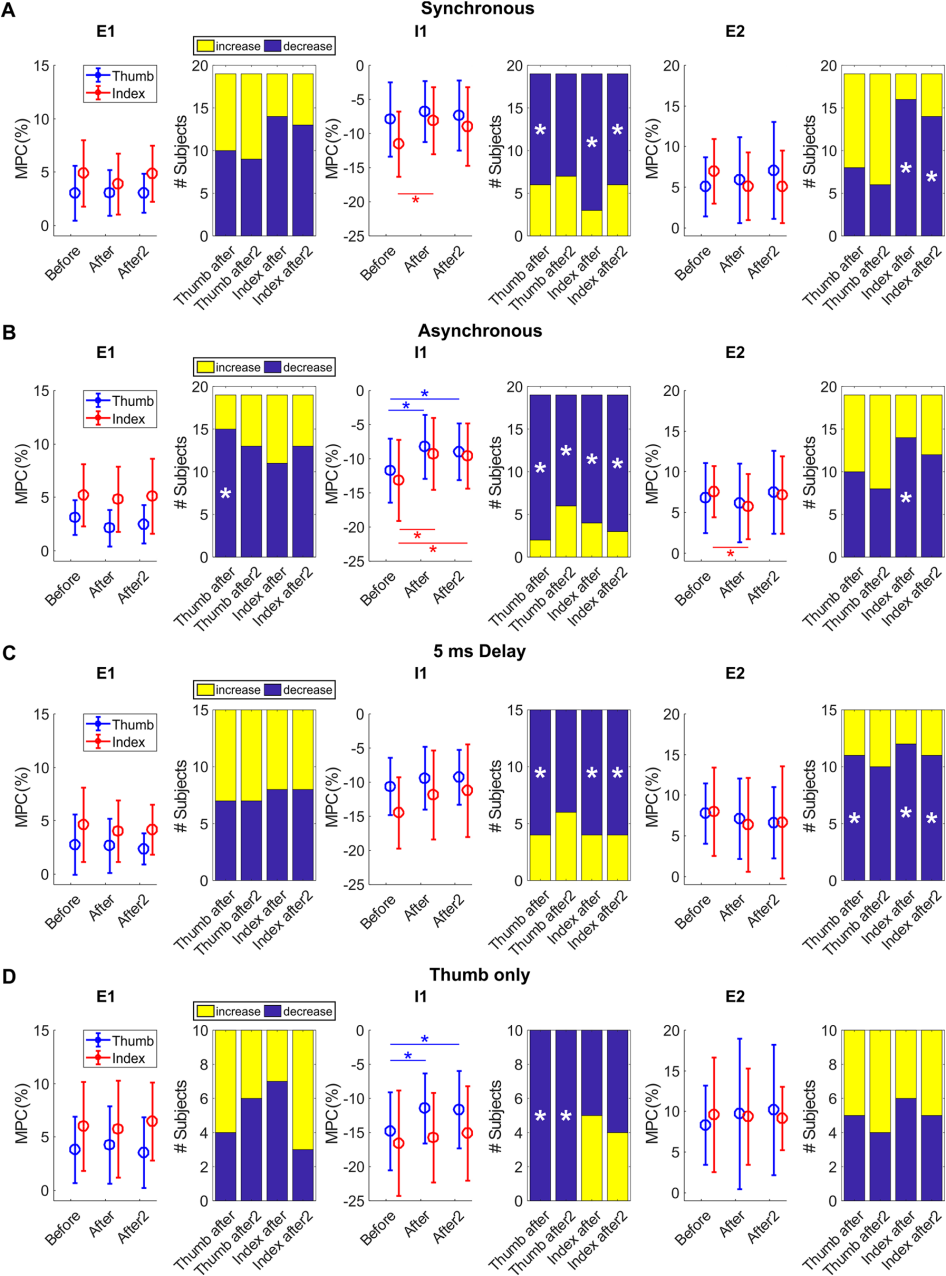
Group results showing MPC for the different stimulation protocols. Mean MPC for E1 (left), I1 (middle), and E2 (right) components of the CMR before, 0–18 min after (after), and 18–32 min after (after2) paired stimulation. Circles represent group mean for thumb (blue) and index (red) fingers; error bars indicate standard deviations. Asterisks indicate significant differences. Colored bars show the number of subjects showing an increase (yellow) or decrease (dark blue) after the stimulation protocol. Asterisks indicate proportions significantly different from the 50% expected by chance. ***A***, Mean MPC and the number of subjects for the synchronous stimulation protocol. ***B***, Mean MPC and the number of subjects for the asynchronous stimulation protocol. ***C***, Mean MPC and number of subjects for the 5 ms delay stimulation protocol. ***D***, Mean MPC and number of subjects for the thumb-only stimulation protocol.

Figure 3 depicts the MPC for each type of paired stimulation (Fig. 3*A*, synchronous; Fig. 3*B*, asynchronous; Fig. 3*C*, 5 ms delay; Fig. 3*D*, thumb only) for each assessment time point (before, after, and after2) of the thumb (blue) and index (red) finger stimulation.

Mauchly’s test of sphericity indicated that the assumption of sphericity was met for the thumb (*χ*^2^_(2)_ = 2.296; *p* = 0.317) but not index (*χ*^2^_(2)_ = 7.818; *p* = 0.020) stimulation.

For both thumb and index finger stimulation, there was no effect of TIME (thumb, *F*_(2,118)_ = 0.770; *p* = 0.465; index, *F*_(1.776, 104.786)_ = 2.036; *p* = 0.141) or CONDITION (thumb, *F*_(3,59)_ = 1.096; *p* = 0.358; index, *F*_(3,59)_ = 0.891; *p* = 0.451) or their interaction (thumb, *F*_(6,118)_ = 0.767; *p* = 0.597; index, *F*_(5.328, 104.786)_ = 0.342; *p* = 0.896) on the size of the E1 reflex component.

However, there was a decrease in E1 in more subjects than expected by chance in response to thumb stimulation after asynchronous stimulation (*p* = 0.002; Fig. 3*B*, left).

#### Decreases in I1 reflex component

The assumption of sphericity was met for both index (*χ*^2^_(2)_ = 4.083; *p* = 0.130) and thumb (*χ*^2^_(2)_ = 3.251; *p* = 0.197) stimulation.

There was a significant effect of TIME (thumb, *F*_(2,118)_ = 13.719; *p* < 0.001; partial, *η*^2^ = 0.1887; index, *F*_(2,118)_ = 13.169; *p* < 0.001; partial, *η*^2^ = 0.1825) and CONDITION (thumb, *F*_(3,59)_ = 3.376; *p* = 0.024; partial *η*^2^ = 0.1465; index, *F*_(3,59)_ = 3.610; *p* = 0.018; partial, *η*^2^ = 0.1551), but not their interaction (thumb, *F*_(6,118)_ = 1.333; *p* = 0.248; index, *F*_(6,118)_ = 0.633; *p* = 0.704) on the size of the I1 reflex component in response to both thumb and index finger stimulation.

Post hoc *t* tests confirmed that I1 decreased in size (i.e., became less negative) in response to index finger stimulation after synchronous stimulation (−11.5 ± 4.8% decreased to −8.1 ± 4.9%; *t*_(18)_ = 4.329; *p* < 0.001; *d* = 0.4358; Fig. 3*A*, middle), though this change just failed to reach significance for the after2 assessment (−9.0 ± 5.8%; *t*_(18)_ = 2.275; *p* = 0.035; threshold for significance *p* < 0.025 using Benjamini–Hochberg correction for multiple comparisons). This decrease for I1 was seen in significantly more participants than the 50% expected by chance, in both the after and after2 assessment, for index finger stimulation (*p* < 0.001 index after and *p* = 0.032 after2), but only in the immediate after assessment for thumb stimulation (*p* = 0.032 thumb after and *p* = 0.084 after2).

The biggest change was seen in I1 after asynchronous stimulation (Fig. 3*B*, middle). Post hoc *t* tests showed a significant reflex decrease in response to both thumb and index finger stimulation, in both after assessments (thumb before, −11.7 ± 4.7%; thumb after, −8.2 ± 4.7%; *t*_(18)_ = 3.607; *p* = 0.002; *d* = 0.4602; thumb after2, −9.0 ± 4.1%; *t*_(18)_ = 2.731; *p* = 0.014; *d* = 0.3741; index before, −13.2 ± 5.9%; index after, −9.3 ± 5.3%; *t*_(18)_ = 3.017; *p* = 0.007; *d* = 0.4417; index after2, −9.6 ± 4.8%; *t*_(18)_ = 3.264; *p* = 0.004; *d* = 0.4228). The number of subjects showing this decrease was also significantly higher than the 50% expected by chance (*p* < 0.001 thumb after and *p* = 0.032 after2; *p* = 0.002 index after and *p* < 0.001 after2).

There were no significant changes in I1 after the 5 ms delay stimulation protocol (Fig. 3*C*, middle). However, more subjects than expected by chance showed a decrease, except for the after2 assessment following thumb stimulation (*p* = 0.018 for thumb after and *p* = 0.151 after2 and *p* = 0.018 for index after and after2).

After the thumb-only stimulation protocol (Fig. 3*D*, middle), the I1 decreased significantly in response to thumb stimulation both in the after and after2 assessments (thumb before, −14.8 ± 5.8%; thumb after, −11.4 ± 5.1%; *t*_(9)_ = 6.907; *p* = 0; *d* = 2.6516; thumb after2, −11.7 ± 5.7%; *t*_(9)_ = 6.032; *p* = 0; *d* = 2.3153). This change could be seen in all subjects of this group (*p* < 0.001 for thumb after and after2). In contrast, no changes in the I1 following index finger stimulation were seen.

#### Limited changes in E2 reflex component

Mauchly’s test of sphericity indicated that the assumption of sphericity was violated for thumb (*χ*^2^_(2)_ = 9.222; *p* = 0.010) but not index (*χ*^2^_(2)_ = 5.844; *p* = 0.054) stimulation.

There was a significant effect of TIME on the E2 component of the CMR but only in response to index finger stimulation (index, *F*_(2,118)_ = 4.142; *p* = 0.018; partial, *η*2 = 0.0656; thumb, *F*_(1.744, 102.877)_ = 1.452; *p* = 0.239). However, there was no effect of CONDITION (thumb, *F*_(3,59)_ = 1.133; *p* = 0.343; index, *F*_(3,59)_ = 1.517; *p* = 0.219) or their interaction (thumb, *F*_(5.231, 102.877)_ = 1.237; *p* = 0.297; index, *F*_(6,118)_ = 0.578; *p* = 0.747) in response to either stimulation.

Post hoc *t* tests showed no significant changes in E2 after paired stimulation, except for a decrease in response to index finger stimulation after asynchronous stimulation (7.5 ± 3.1% decreased to 5.7 ± 4.0%; *t*_(18)_ = 3.425; *p* = 0.003; *d* = 0.2798; Fig. 3*B*, right). More subjects than the 50% expected by chance showed a decrease in E2 in response to index finger stimulation after synchronous, asynchronous, and the 5 ms delay stimulation protocols, apart from the second after assessment following asynchronous stimulation (synchronous index, *p* < 0.001 after and *p* = 0.010 after2; asynchronous index, *p* = 0.010 after and *p* = 0.084 after2; 5 ms delay index, *p* = 0.004 after and *p* = 0.018 after2). In response to thumb stimulation, this was only seen in the 5 ms delay protocol, though the after2 assessment just misses significance (*p* = 0.018 thumb after and *p* = 0.060 after2).

### Reflex summation

**Figure 4.**
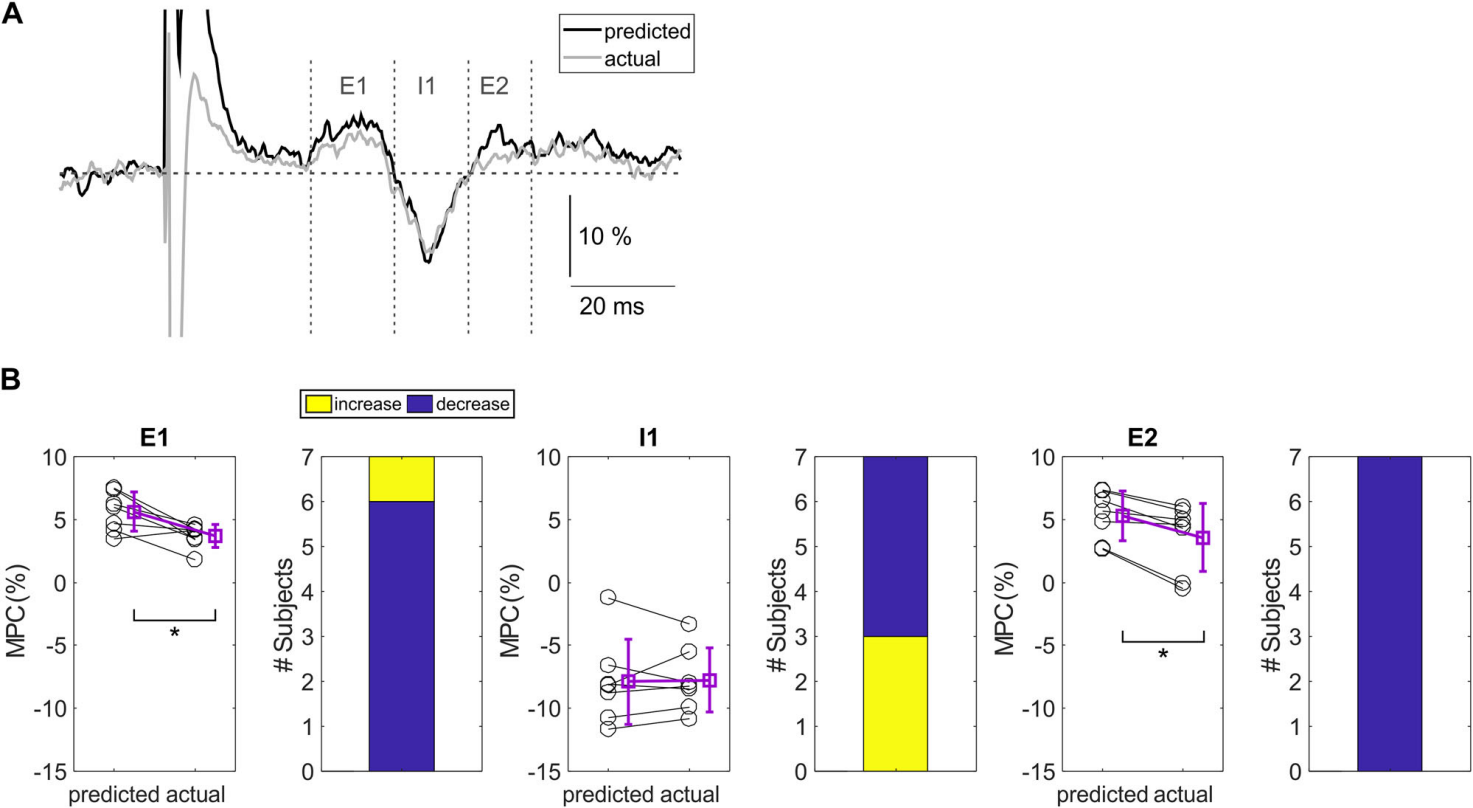
Group results for the reflex summation experiment. ***A***, Overlay of EMG traces averaged across all subjects, for the predicted (black) and the actual response (gray). Gray vertical dotted lines indicate the three components (E1, I1, E2) of the cutaneoumuscular reflex. Gray horizontal dashed line represents the mean baseline value of the average actual response trace. Calibration bar is a percentage of the baseline EMG level. ***B***, Mean MPC for E1 (left), I1 (middle), and E2 (right) components of the CMR for the predicted response and the actual response. Single subjects are shown in black; group mean is shown in purple. Error bars indicate standard deviations. Asterisks indicate significant differences. Colored bars show the number of subjects showing an increased (yellow) or decreased (dark blue) MPC for the actual response compared with the predicted response.

Figure 4 depicts the group results for the reflex summation experiment. Figure 4*A* shows the average predicted and actual responses, averaged across all subjects. The predicted trace has been constructed assuming linear summation between the two stimuli; the actual trace shows the actual response to paired index finger and thumb stimulation, with a correction to ensure the baseline is the same as the predicted trace (see full description of calculation in Materials and Methods). Both E1 and E2 appeared to be slightly reduced in the actual traces, while I1 was comparable between the two.

When comparing the predicted response with the actual response quantitatively (Fig. 4*B*), paired *t* tests showed that both excitatory reflex components were significantly reduced in the actual response. For E1, the actual response (3.7 ± 0.9%) was smaller compared with the predicted response (5.6 ± 1.6%) in six out of seven subjects (*t*_(6)_ = 3.193; *p* = 0.019; *d* = 0.6906). For E2, the actual response (3.6 ± 2.7%) was smaller compared with the predicted response (5.3 ± 2.0%) in all subjects (*t*_(6)_ = 4.168; *p* = 0.006; *d* = 0.4429). There was no difference between predicted (−7.9 ± 3.4%) and actual (−7.7 ± 2.6%) response for the I1 component of the CMR (*t*_(6)_ = −0.234; *p* = 0.822).

These results suggest that paired thumb and index finger stimulations do not sum linearly and in fact lead to a reduction of E1 and E2.

## Discussion

Delivering paired stimulation often results in long-lasting, plastic changes in neural circuits responding to the stimuli. Where plasticity is dependent on precise stimulus timing, the underlying cellular mechanism is likely to be STDP (Markram et al., 1997). The operation of STDP is straightforward to understand when one stimulus accesses synaptic inputs to a cell population, and the other triggers action potentials. For example, TMS delivered to the motor cortex activates corticospinal inputs to motoneurons, and supramaximal stimulation of a peripheral nerve sets up antidromic action potentials which generate motoneuron spiking. By appropriately timing these stimuli, the conditions for STDP can be met and plasticity can be induced (Taylor and Martin, 2009). Alternatively, STDP may also be induced by activating different inputs which converge onto a common target cell population. In this case, the convergent input will elicit response spikes more often than a single input would; this can skew the balance between synaptic potentiation and depression and lead to net strengthening of the synapse (Brown et al., 2016). Such a mechanism seems to be the basis of several paired associative stimulation approaches (Stefan et al., 2000; Ridding and Uy, 2003). However, it will only be effective if the paired stimuli converge onto overlapping neural populations and if the timing of spikes relative to the synaptic inputs that elicit them becomes more reliably timed.

Paired associative simulation protocols use a wide range of stimulation duration. Greater effects can be seen by delivering longer durations of paired stimuli (Grover et al., 2023). We therefore need to consider whether the failure to find plasticity in the CMR here was due to using insufficient paired stimuli. In some previous work, paired stimulation was used for 1 h or more (Ridding et al., 2000; McKay et al., 2002; Charlton et al., 2003), sometimes even as long as 7 h (Brown et al., 2016). However, long-term plasticity has been successfully induced with much shorter paired stimulation durations (Stefan et al., 2000; Ziemann et al., 2004; Cirillo et al., 2009; Player et al., 2012). Stefan et al. (2000) and Cirillo et al. (2009) found changes lasting at least 30–60 min after just 90 pairings given over 30 min. Under some specific conditions, induction of long-term potentiation has been shown to require only a few stimuli, to the extent that even a single conditioning stimulus may suffice (Maren et al., 1994). These studies demonstrate that plasticity induced by paired stimulation can evolve rapidly and be persistent. This is further backed up by animal studies which found structural changes associated with plasticity developed within 30 min (Engert and Bonhoeffer, 1999). In this study, we used 18,000 paired stimuli delivered over 30 min. It seems unlikely that different results would be obtained using even more stimuli, but we cannot exclude this possibility.

In this report, we have demonstrated that paired stimulation of two digits does not lead to potentiation of the different components of the CMR. Comparison of the responses to stimuli given alone versus both together suggested slightly sublinear summation of E1 and E2 components—the combined response was a little smaller than expected from the linear sum of each response alone. A similar observation was made by Matthews (1993), who noted that larger stretches of the wrist elicited larger stretch reflexes, but the response increased far less than simple proportion. He concluded that this may arise from an interference between short- and long-latency excitatory reflexes initiated by the same stimulus; the exact nature of the summation differs by muscle (Matthews, 1994). In the CMR, sublinear summation is consistent with some saturation in the underlying pathways. It has been reported in animal studies that cells in both the spinal cord and cuneate nucleus fire a powerful burst of action potentials in response to a single cutaneous stimulus (Gregor and Zimmermann, 1972; Witham and Baker, 2011); such responses may have little chance of augmentation by further convergent input. This would mean that induction of plasticity after paired stimuli was no more likely than after a single stimulus, when presumably homeostatic mechanisms serve to maintain synaptic efficacy reasonably constant.

An alternative explanation for the lack of plasticity in the CMR is that inputs from the two digits are processed in independent channels; without convergence, there would then be no opportunity for paired stimuli to raise the probability of postsynaptic cell discharge and make a spike response following synaptic input more likely. However, this possibility appears less likely. Responses to paired stimuli showed significantly sublinear summation, which is only explicable if there was some overlap in responding populations. Additionally, some cells within the cuneate nucleus (Witham and Baker, 2011) and motor cortex (Asanuma and Rosen, 1972) are known to receive input from wide receptive fields.

A further possible explanation for our results is that the circuits conveying cutaneous information from the digits are simply not susceptible to plasticity. There is no reason why all circuits should be equally plastic: this laboratory has previously shown, for example, that motor circuits controlling extensor muscles show markedly less plasticity than those for flexors (Foysal and Baker, 2019). It is known that paired motor point stimulation of two hand muscles can induce plasticity in cortical responses (Ridding and Uy, 2003); perhaps circuits processing sensory input from muscle show more plasticity than those involved in cutaneous sensation. We cannot rule this possibility out. However, after stroke when the supraspinal components (I1/E2) of the CMR are lost, the E1 component increases (Nadler et al., 2004). In contrast, during motor learning, the I1 and E2 components can increase (Nadler et al., 2000). These results indicate that the CMR is capable of showing long-term plastic changes under the right conditions.

The only long-term change in the CMR which we observed was a consistent reduction in the I1 component. However, this did not occur only when paired stimuli were delivered at a timing intended to induce plasticity. Rather, significant I1 reductions were seem in all stimulus conditions observed, even when a single input was stimulated alone. This change is most likely to reflect fatigue or synaptic depression consequent on repeated activation, rather than a plastic process.

## Synthesis

Reviewing Editor: Eran Perlson, Tel Aviv University Decisions are customarily a result of the Reviewing Editor and the peer reviewers coming together and discussing their recommendations until a consensus is reached. When revisions are invited, a fact-based synthesis statement explaining their decision and outlining what is needed to prepare a revision will be listed below. The following reviewer(s) agreed to reveal their identity: Thomas Richards.

This manuscript presents the interesting finding that paired stimulation of two digits does not produce long-term plastic changes in the CMR. If true, this represents a significant finding, since paired stimulation does induce long-term plastic changes in other neural pathways. There are implications for informing how therapy is delivered for conditions such as stroke and SCI, since paired stimulation of convergent afferents can produce long-term changes in excitability with high muscle specificity. Generally, the manuscript is well written. It has a clear aim, which it then addresses with appropriate methodology. The results are described appropriately. The figures are clear and relevant. The discussion is focused and concise. Still, a potential methodological limitation of the current study is that the total duration that conditioning stimuli delivered may not have been long enough to induce long-term plastic changes. This should be acknowledged.

The investigators explored whether 4 stimulation protocols of two digits (thumb and index) had long-term effects on the CMR, recorded in the FDI. Assessments of the CMR were carried out before stimulation, and at two time points shortly after stimulation. Since the authors saw no evidence of long-term plasticity with paired stimulation, they compared the sum of the separate CMR responses from each digit to a paired response to know whether they summed linearly. They report that the sum is sub-linear, suggesting response saturation with a single input, and propose three potential mechanisms for why no long-term plasticity was seen.

As most previous studies that observed long-term plastic changes delivered conditioning stimuli for at least double, and commonly for more than 3 times the total time used in the current study, the methodological differences between need to be acknowledged, considering the potential impact of not stimulating for long enough on the main finding of the study.

Specific comments

Line 126: "...subjects were conditioned with one of the four paired stimulation protocols described below, each of which lasted approximately 30 minutes..."

The authors cite several studies whereby stimulation of peripheral nerves has induced plasticity in the motor cortex and corticospinal projections (Ridding et al., 2000, 2001; McKay et al., 2002; Charlton et al., 2003), and also cite Brown et al. (2016). In all of these studies, the duration of paired stimulation was 1h or more, and even for as long as 7h before it induced plastic changes (Brown et al., 2016). Mckay (2002) specifically looks at the time course of excitability changes with regards to stimulation of afferent nerves. In this study, no excitability changes were seen until 45 mins after stimulation began, and this is during a 1 min period between stimulation delivery. In primates, 1 h of paired median and ulnar stimulation was required to produce cortical changes (Habekost, Germann and Baker, 2021). You cite (Ridding and Uy, 2003), commonly referred to elsewhere for methods, who give associative or non-associative stimulation for 1 h.

Though there are some contextual differences between the studies above and the present study, to expect long-term plastic changes, at least 45 mins of stimulation or even an hour of stimulation would have been safe. However, the current study only provided conditioning stimuli for 30 mins. Considering the conclusions of the current study, this methodological difference is significant since it has the potential to have major implications on the outcome of the study.

Line 134: "5 ms Delay."

What specifically is the purpose of this condition?

Line 142: "Mean percentage increase (MPI) was determined as... "

If the change is relative to baseline, wont positive values indicate an increase and negative values a decrease? If this is the case, perhaps Mean Percentage Change would be a less confusing way to refer to this metric considering the fact that there are cases where MPI can be negative or all subjects show a decrease (I1 Fig. 3D).

Line 295: "This is consistent with some saturation in the underlying pathways."

Considering the different assumptions and ways in which EMG can sum (discussed in (Baker and Lemon, 1995)), I think it would be useful if you could provide a reference which illustrates a similar situation where this is the case.

The authors propose three potential reasons for lack of long-term plasticity: a putative homeostatic mechanism, a lack of convergence, and lack of susceptibility to plasticity in this pathway. To me, lack of convergence seems unlikely considering the sum, but should still be mentioned. The two remaining two explanations seem possible.

Line 449: Figure 4.

Since the magnitude differs considerably from the first experiment, it would be useful if you could provide average traces for this sample for the thumb/finger stimulation only for this experiment.

Final comments

This is an interesting study that presents negative findings and a follow-up experiment to explore the reasons for this. The statistics are appropriate, as are the methods (with the exception of the duration of stimulation), and the figures showing changes in the CMR components display the direction of change across the sample, which is very helpful for interpreting the results. Given the potential implications for the main finding, the duration of stimulation should be mentioned in the title, e.g.,:

"Paired Stimulation of Different Digits for 30 Minutes Does Not Produce Long-Term Plastic Changes in the Human Cutaneomuscular Reflex"

Also, there should be a paragraph in the discussion to acknowledge the methodological differences and significance of exposure to stimulation. It may be that the authors are aware of studies in which long-term changes have been seen after a shorter time, in which case they could provide these.

References (if not cited in manuscript)

Habekost, B., Germann, M. and Baker, S.N. (2021) 'Plastic changes in primate motor cortex following paired peripheral nerve stimulation', Journal of Neurophysiology, 125(2), pp. 458-475. Available at: https://doi.org/10.1152/jn.00288.2020.
